# The Nuanced Metabolic Functions of Endogenous FGF21 Depend on the Nature of the Stimulus, Tissue Source, and Experimental Model

**DOI:** 10.3389/fendo.2021.802541

**Published:** 2022-01-03

**Authors:** Redin A. Spann, Christopher D. Morrison, Laura J. den Hartigh

**Affiliations:** ^1^ Pennington Biomedical Research Center, Louisiana State University System, Baton Rouge, LA, United States; ^2^ Department of Medicine, Division of Metabolism, Endocrinology and Nutrition, University of Washington, Seattle, WA, United States; ^3^ Diabetes Institute, University of Washington, Seattle, WA, United States

**Keywords:** brain, liver, protein restriction, obesity, fasting, adipose tissue, cold exposure

## Abstract

Fibroblast growth factor 21 (FGF21) is a hormone that is involved in the regulation of lipid, glucose, and energy metabolism. Pharmacological FGF21 administration promotes weight loss and improves insulin sensitivity in rodents, non-human primates, and humans. However, pharmacologic effects of FGF21 likely differ from its physiological effects. Endogenous FGF21 is produced by many cell types, including hepatocytes, white and brown adipocytes, skeletal and cardiac myocytes, and pancreatic beta cells, and acts on a diverse array of effector tissues such as the brain, white and brown adipose tissue, heart, and skeletal muscle. Different receptor expression patterns dictate FGF21 function in these target tissues, with the primary effect to coordinate responses to nutritional stress. Moreover, different nutritional stimuli tend to promote FGF21 expression from different tissues; i.e., fasting induces hepatic-derived FGF21, while feeding promotes white adipocyte-derived FGF21. Target tissue effects of FGF21 also depend on its capacity to enter the systemic circulation, which varies widely from known FGF21 tissue sources in response to various stimuli. Due to its association with obesity and non-alcoholic fatty liver disease, the metabolic effects of endogenously produced FGF21 during the pathogenesis of these conditions are not well known. In this review, we will highlight what is known about endogenous tissue-specific FGF21 expression and organ cross-talk that dictate its diverse physiological functions, with particular attention given to FGF21 responses to nutritional stress. The importance of the particular experimental design, cellular and animal models, and nutritional status in deciphering the diverse metabolic functions of endogenous FGF21 cannot be overstated.

## 1 Introduction

Fibroblast growth factor 21 (FGF21) is a hormone that beneficially regulates glucose and lipid metabolism and contributes to energy balance. As such, FGF21 has been extensively studied as a pharmacological agent that promotes weight loss, increases energy expenditure, and improves insulin sensitivity in animal models and humans (1–7). Many studies have reported beneficial effects of pharmacological FGF21 administration, including improvements in hyperglycemia, insulin resistance, and dyslipidemia, reduced fat mass, and reversal of non-alcoholic steatohepatitis (NASH) [reviewed in (8, 9, and (10)]. Despite benefits to energy metabolism in rodent and non-human primate models, clear beneficial FGF21 pharmacology in humans has been difficult to achieve. For reasons that are still unknown, the glucose-lowering effects of pharmacological FGF21 are absent or less pronounced in humans (5, 7, 11), while its effects on dyslipidemia are highly conserved between rodents and humans (8).

Unlike other canonical FGFs with a heparin binding domain that limit endocrine activity, such as FGF1, the structure of FGF21 lacks this key heparin binding domain, and thus more closely resembles other endocrine growth factor family members such as FGF19 (and the mouse orthologue FGF15) and FGF23, which allows it to circulate in an endocrine manner. In order to act on target tissues, endocrine FGFs bind to several FGF receptors (FGFRs) and a required coreceptor, either α-klotho or β-klotho [reviewed in (12)]. FGFR1-4 are highly conserved across a wide array of mammalian species and vary in their ligand affinity and tissue distribution, enabling a diversity of effects from endocrine FGFs. FGF21 specifically binds to FGFR1 with the highest affinity in the presence of β-klotho (KLB), which triggers autophosphorylation of FGFR1 and subsequent activation of the MAPK pathway leading to phosphorylation of ERK1/2 (13), widely used as a surrogate for FGF21-mediated signaling. However, conclusive evidence that FGF21-mediated physiological effects *require* ERK1/2 activation are lacking, and it is not yet established that intracellular signaling pathways initiated by the activation of the FGFR1/KLB complex are the same in all FGF21 target tissues (14). Later sections will describe emerging and varied signaling pathways mediated by FGF21 in particular tissues.

While the pharmacological benefits of FGF21 administration are noteworthy (at least in rodents), they have been reviewed extensively elsewhere and will not be presented in this review. However, some evidence suggests that the pharmacological effects of FGF21 may differ from its physiological effects (15). FGF21 is expressed and often secreted from various tissues, including the liver, white adipose tissue (WAT), brown adipose tissue (BAT), pancreas, and skeletal muscle in response to fasting, feeding, protein restriction, cold exposure, and exercise. Importantly, the tissue origins of FGF21 depend entirely on the nature of the stimulus. For example, fasting and a ketogenic diet increase hepatic FGF21 expression (16–18). Conversely, FGF21 derives from WAT and pancreas during overfeeding and obesity-inducing conditions (19–21), from BAT during cold exposure (22–24), and from skeletal muscle during exercise (25). Collectively, it appears that conditions requiring the mobilization of energy stores induce hepatic and BAT-derived FGF21, while conditions that promote energy storage induce WAT and pancreatic FGF21.

In addition to the complexity of FGF21 expression kinetics in response to different dietary or environmental stressors, FGF21 levels are paradoxically elevated in individuals with obesity (3, 26–29), non-alcoholic steatohepatitis (NASH) (30), and non-alcoholic fatty liver disease (NAFLD) (31, 32). One working theory is that obesity-associated increases in FGF21 reflect an FGF21-resistant state due to observed downregulation of FGFR1c and KLB in the liver and WAT, as well as reduced efficacy of recombinant FGF21 (rFGF21) in mice and humans (33, 34). However, the concept of FGF21 resistance has been subsequently challenged (35–37), discussed in more detail in Section 3.2.4. Another theory posits that dysfunctional white adipocytes, which are abundant in the obese condition, could secrete FGF21 (38). In addition, current consensus suggests that elevated FGF21 observed in NASH is in response to metabolic stress. Nevertheless, a clear consensus regarding the physiological role (if any) played by the increased FGF21 observed in obesity and/or NASH is still lacking. The purpose of this review is to critically examine and discuss the various physiological stimuli for endogenous FGF21, with particular attention given to the nuanced methodology and experimental models employed in studies to date. In particular, a key emphasis is the concept that multiple metabolic tissues produce FGF21, but that they appear to differentially respond to various physiological and pathophysiological stimuli and vary in their contribution to systemic vs. local (i.e. autocrine) FGF21 effects.

## 2 Regulation of FGF21 Gene and Protein Expression and Secretion

**Figure 1:**
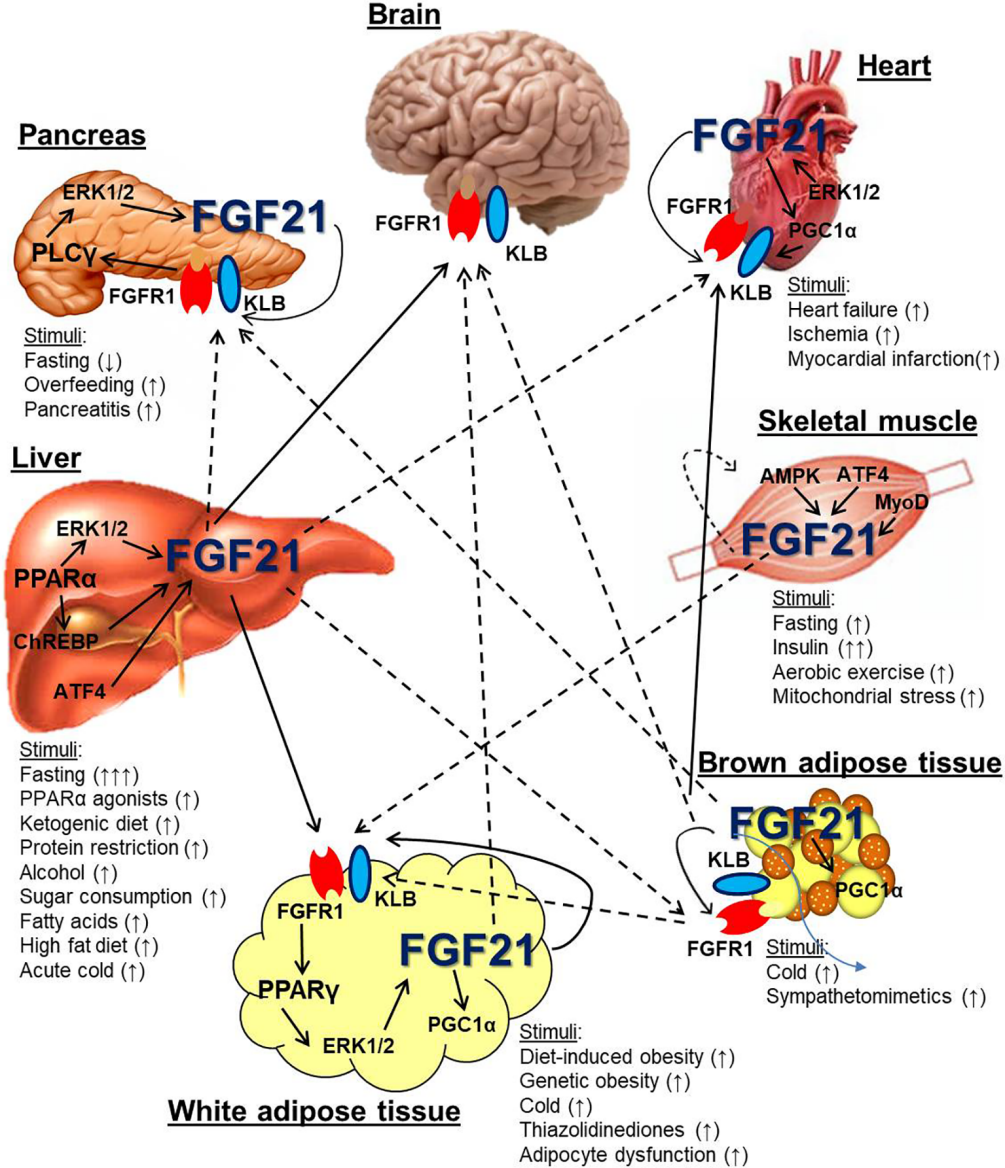
Metabolic tissue endogenous FGF21 expression and cross-talk. Several metabolic organs, including the liver, white and brown adipose tissue, skeletal muscle, pancreas, and heart express and secrete FGF21 in response to various stimuli. The liver is a major source of systemic FGF21, which can target the FGFR1/KLB complex in the brain and white adipose tissue (solid arrows). It is also hypothesized that liver-derived FGF21 can signal directly to the pancreas, heart, and brown adipose tissue (dotted arrows). Skeletal muscle and brown adipose tissue can also express FGF21 that may also circulate. Other organs, including white adipose tissue, pancreas, and heart, also express FGF21, which likely serves an autocrine/paracrine function and has not conclusively been shown to circulate. Dotted arrows indicate the potential for FGF21 from white and brown adipocytes to circulate under particular metabolic conditions such as cold exposure and obesity.

FGF21 mRNA and protein is expressed from a variety of cell types in response to many physiological stimuli (see **Table 1**), but the primary tissues known to express FGF21 are the liver, adipose tissue, pancreas, and skeletal muscle (98) (see **Figure 1**). The propensity for a particular tissue to secrete endocrine FGF21 also differs considerably by tissue type and stimulus. Herein, we will discuss what is currently known about FGF21 derived from these and other tissues that have been less extensively studied, such as cardiac muscle.

**Table 1:** Stimuli for tissue-specific FGF21 expression.

Tissue	Stimulus	Experimental model		Contributes to systemic FGF21?
		Animals and cultured cells	Humans	
**Liver**	Fasting	12-24 hr fast in mice (16–18, 39–42).	7-10 d fast in humans (43, 44).	Yes
	PPARα agonists	PPARα KO mice, human and mouse hepatocytes, *ob/ob* mice (16–18, 40).	Human hepatocytes (17, 18), humans given PPARα agonists (43, 45).	Yes
	Protein restriction	Rats and mice fed a LP diet for 1-21 d (46–50), 7-12 wk (51–53), or 67 wk (48); mice fed a leucine-deficient diet for 7 d (54); mice fed a methionine-restricted diet for 7-11 wk (55–58); amino acid-starved HepG2 cells (54).	Humans consuming a LP diet for 1-43 d (46, 49, 59).	Yes
	Ketogenic diet	7-30 d (16, 46, 60, 61) or 8-9 wk KD (53, 62) in mice.		Yes
	Alcohol consumption	Mice consuming ethanol chronically (63–65).	Humans consuming ethanol acutely (63) or chronically (65).	Yes
	Simple sugar consumption	Fructose, dextrose, and glucose consumption in mice (40, 66, 67), glucose-stimulated rat hepatocytes or HepG2 cells (68, 69).	Fructose and glucose consumption in humans (66, 70), consumption of a carbohydrate-rich diet for 3 days in humans (20).	Yes
	Fatty acids	Oleic and linoleic acid in HepG2 cells (71).	Lipid infusion in humans (71).	Unknown
	High fat diet	C57Bl/6 mice fed HFD for 10 wk (35, 72, 73).		Potentially
	Acute cold	C57Bl/6 mice exposed to 4°C for ≤6 hr (74).		Potentially
**WAT**	Obesity (genetic or DIO)	C57Bl/6, *ob/ob*, *db/db* mice (3, 28, 35, 72, 73, 75), non-human primates (29).	Humans with obesity (3, 26–28, 76–78).	No
	Cold exposure	Mice exposed to 4°C for ≤24 hr (23, 74), 1d (79), or 28 d (24).		No
	TZDs	3T3-L1 adipocytes treated with rosiglitazone (10 μM for 24 h) (72, 80); C57Bl/6 and db/db mice given rosiglitazone (30-100 mg/kg for 8 d) (72, 81).		Potentially
	Adipocyte dysfunction	Mice overexpressing ferritin from adipocytes (38).		Yes
**BAT**	Cold exposure	Mice exposed to acute cold (4-8 hr at 4°C) (22, 82), and chronic cold (1-30 d) (23, 24, 79).	Humans exposed to cold for 12 hr (83).	Yes/no
	Sympathomimetics	Cultured brown adipocytes treated with NE and Iso (23, 79); mice given CL316243 or Iso IP (22, 79).		Potentially
**Pancreas**	Overfeeding	Mice in the fed state (81, 84) or fed a HFD for 16 wk (21).		No
	Cerulein-induced pancreatitis	Mice injected with cerulean (50 μg/kg) for 4 hr (85).		No
**Heart**	Heart failure		Humans undergoing heart transplant due to heart failure (86).	Unknown
	Ischemia	Mice with coronary artery ligation-induced MI (87).	Human subjects admitted for MI (87, 88).	Potentially
	Hypertrophy	Mice treated with isoproterenol/phenylephrine (89).		Potentially
**Skeletal muscle**	Aerobic exercise	Mice acutely and chronically running on wheels (25).	Healthy men using exercise bicycles for 1 hr (25), treadmill for ≤ 1 hr (90–92).	Yes
	Fasting	Mice fasted for 48 hr (93).		Unknown
	Insulin		3-4 hr insulin infusion in healthy men (94).	Unknown
	Mitochondrial stress	Mice with skeletal muscle mitochondrial dysfunction (95); mice overexpressing PLIN5 (96).	Adults and children with skeletal muscle mitochondrial disorders (97).	Potentially

BAT, brown adipose tissue; CL316243, β_3_ adrenergic receptor agonist; d, days; DIO, diet-induced obesity; HepG2, hepatocyte cell line; HFD, high fat diet; hr, hours; IP, intraperitoneal; Iso, isoproterenol; KD, ketogenic diet (high fat, low carb); KO, knock out; LP, low protein diet; MI, myocardial infarction; NE, norepinephrine; ob/ob, leptin-deficient mice; PLIN5, perilipin 5; PPARα, peroxisome proliferator-activated receptor alpha; TZDs, thiazoladinediones; WAT, white adipose tissue; wk, weeks.

### 2.1 Stimuli for Endogenous Endocrine Hepatic FGF21 Release

Liver-derived FGF21 has been extensively studied under many nutritional conditions, including fasting (17, 39), ketogenic diet feeding (16), protein restriction (46), carbohydrate-rich diets (68, 70), and alcohol consumption (63). Several signaling mechanisms for hepatic FGF21 have been described. Fasting-induced hepatic FGF21 is mediated by peroxisome proliferator-activated receptor alpha (PPARα), a transcription factor that is intimately involved with lipid homeostasis (18). Fenofibrates, a class of drugs that are strong PPARα agonists, strongly induce FGF21 gene expression in the liver in mice, while mice deficient in PPARα do not exhibit changes in hepatic FGF21 expression following fasting or PPARα agonism (16). Long-term fasting (i.e. greater than 12 hours), a ketogenic diet, and free fatty acids have been shown to stimulate such PPARα-mediated hepatic FGF21 production (16, 17, 71). However, additional dietary perturbations that increase hepatic FGF21 including excess glucose or fructose intake have more recently shown a dependence on the carbohydrate response element binding protein (ChREBP), in addition to PPARα (40, 66, 67). Another study reports the presence of both carbohydrate and glucagon response elements in the FGF21 promotor region that enable its activation by both fed and fasted signals (69). In later subsections, various stimuli for hepatic FGF21 will be discussed.

The first group to generate a global FGF21 knock out (KO) loss-of-function mouse model reported that these mice had increased body weight with more fat and lean mass and impaired glucose tolerance compared to wild-type (WT) mice fed a chow diet (99), a phenotype that has been replicated in other FGF21 KO mice (100). A subsequent and different FGF21 KO mouse model did not display increased body weight over time, but had a higher percentage of body fat due to lower energy expenditure (101). Once it became apparent that FGF21 is expressed and secreted from multiple metabolic tissues, conditional knock out mice were generated in which FGF21 expression was limited from either the liver or adipose tissue (to be discussed in later sections). Such complex loss-of-function models continue to be developed to increase our understanding of the source-dependent metabolic effects of FGF21.

The first gain-of-function mouse model used to study specific effects of hepatic FGF21 (Hep-FGF21) utilized a transgenic strategy in which FGF21 was expressed under the control of the ApoE promoter (102). ApoE is primarily expressed from hepatocytes, but is also known to be expressed from other cell types such as macrophages, suggesting a model of FGF21 expression that is not entirely liver-specific. Hepatic FGF21 (Hep-FGF21) expression levels were 2.5 times higher in Hep-FGF21^Tg^ mice, which led to lower body weight, lower fasting glucose, improved glucose and insulin tolerance, reduced liver fat, more brown adipose tissue, and smaller subcutaneous adipocytes than in wild-type control mice. When challenged with a high fat diet (HFD), Hep-FGF21^Tg^ mice consumed more food, yet gained less body weight than WT controls (102). A subsequent Hep-FGF21^Tg^ mouse model (with FGF21 expression similarly driven by the ApoE promoter) enhanced hepatic FGF21 expression by 50-fold under fasted conditions, and effectively reduced plasma cholesterol, glucose, and insulin levels while increasing expression of WAT lipolysis markers including hormone sensitive lipase (HSL) and adipose triglyceride lipase (ATGL) (17), suggesting that augmented hepatic FGF21 production induces WAT lipolysis. This second Hep-FGF21^Tg^ mouse also revealed a 5-fold induction of peroxisome proliferator-activated receptor gamma coactivator-1 alpha (PGC1α) in the liver (41), which is now thought to represent a major downstream signaling pathway for FGF21.

#### 2.1.1 Fasting

Fasting potently increases FGF21 levels in the blood in rodents, non-human primates, and humans (78). However, the kinetics and degree of fasting required to elicit FGF21 secretion vary widely between species. Mice fasted for as few as 6 hours show marked increases in circulating FGF21 levels (16, 41, 42). By contrast, it takes adult humans at least 60 hours of fasting to achieve a measureable increase in plasma FGF21 (43, 44); however, some studies utilizing extreme fasting in humans have not observed changes in FGF21 levels (31, 45, 103). Nevertheless, this vast interspecies difference in FGF21 kinetics modulated by fasting raises the question of whether the emerging functions of FGF21 in the mouse can be readily applied to humans.

Fasting-induced FGF21 has been consistently shown to be liver-derived in rodent models (39), and is likely to be liver-derived in humans as well (78). Fasting-induced FGF21 has been shown to be regulated by PPARα in mice, with subsequent phosphorylation of ERK1/2 (16, 17, 42). Initial global FGF21^-/-^ mice were found to tolerate acute fasting, but lost less body weight than WT mice following a 24 hour fast (99), suggesting an important role for FGF21 in the mobilization of energy stores in the face of nutritional deficits. Subsequent studies in mice have confirmed that fasting-induced FGF21 derives from the liver (16, 17, 39). Mice with liver-specific FGF21 deletion, generated by crossing FGF21^fl/fl^ mice with Albumin-Cre^Tg^ mice (Hep-FGF21 KO mice), had abolished circulating FGF21 levels upon fasting, and exhibited moderately worsened glucose tolerance than WT mice when fed a chow or HFD (39).

Importantly, most studies that examine hepatic FGF21 use models with moderate to extreme fasting prior to blood and tissue sampling. This becomes extremely important when the sources of FGF21 are then assumed to be exclusively liver-derived, because fasting is known to induce Hep-FGF21. Thus, experimental models that fast animals prior to sampling could be missing minor but potentially biologically meaningful contributions from other tissues.

#### 2.1.2 Alcohol Consumption

Hepatic FGF21 is also induced by acute and chronic alcohol consumption in mice (60, 64) and in humans (63, 65), but it is not currently known whether this is similarly dependent on PPARα or ChREBP. Interestingly, the increased FGF21 induced by chronic alcohol consumption in mice was shown to be protective against significant alcohol-induced liver pathology and mortality (63, 64). Transgenic overexpression of hepatic FGF21 or infusion with rFGF21 reduced the animal’s preference for alcohol over water, while FGF21 deficiency increased alcohol preference (104), suggesting a critical liver-brain FGF21 signaling axis, likely by modulating dopamine release by neurons in the nucleus accumbens (104), a region that controls reward behavior. To date, the only human genome-wide association studies (GWAS) studies that have identified single nucleotide polymorphisms (SNPs) in *KLB* have been in association with alcohol consumption (105–108).

#### 2.1.3 Protein Restriction

Studies have long since indicated that total energy restriction induces an increase in circulating FGF21 in both humans and rodents (17, 41). In humans results have not been consistent, with FGF21 rising in only some studies of food restriction (43), including a prolonged 10-day fast (44), while not changing in other studies (31, 45, 103). Despite these conflicting results, convincing evidence points towards dietary amino acid restriction as being a key trigger of the FGF21 response in the liver (54). Laeger et al. then demonstrated that total protein restriction, and not energy restriction per se, led to increased FGF21 in plasma of both rodents and humans, and that energy expenditure increased while body weight was reduced (46). These results have since been replicated many times by this lab and others (47, 59, 109–112). Also GWAS studies in humans have found associations between *FGF21* alleles and lower protein intake (113, 114). In a study comparing 25 different mouse diets with varying ratios of protein, carbohydrates, and fat as well as total calories, it was determined that low protein content elicited the strongest expression of FGF21 (48). Furthermore, FGF21 signaling in the context of protein restriction decreased body weight and improved glucose handling in diet-induced obese animals (51, 115). While these past studies have shown that a diet of 5% protein is sufficient to reliably increase FGF21, a recent study by Wu and colleagues went as far as decreasing protein content to 1%, and these very low protein diets also led to decreased body weight and improved glucose handling with increased FGF21 expression (52). Perhaps not surprisingly, then, FGF21 seems to signal for the animal to seek out and prefer diets that are high in protein whether protein deprived (51) or not (116) thereby allowing the animal to adapt to the malnutrition.

Attempts to identify discrete amino acids responsible for FGF21 induction have had mixed results. One possibility is that essential amino acids which are preferentially found in animal-based protein sources are responsible for the physiological response observed during protein restriction. Branched-chain amino acids (BCAAs), including the essential amino acids leucine, isoleucine, and valine, have garnered considerable attention due the fact that circulating BCAA levels are positively correlated with a diabetes phenotype (117, 118) and restricting BCAAs have been found to improve health and increase FGF21 (49, 119). A recent study by MacArthur et al. found that while the amino acid profile of animal and plant-based diets differed, FGF21 induction was equal during low-protein (2% and 6%) diet feeding in mice fed purified diets matching these two profiles (120). This suggests that FGF21 is secreted in response to low total dietary protein content, but not to differences in individual amino acids such as lower glutamic acid in animals and lower methionine and lysine in plants. Yu and colleagues restricted each individual BCAA in mice, and found that eliminating isoleucine alone had metabolic benefits and that FGF21 was elevated and at least partially mediated these effects (115). In another study, however, restriction of threonine or tryptophan conferred metabolic response and increased FGF21 (50) while isoleucine did not. De Sousa-Coelho et al. showed that leucine restriction also leads to FGF21 production and the subsequent increase in lipolysis and decrease in lipogenesis (54, 121). Others have also found that methionine restriction alone induces FGF21 and increases energy expenditure (55, 56, 122, 123). Studies now have confirmed that methionine restriction promoted weight and fat loss and increased energy expenditure while increasing systemic FGF21 in mice with obesity, but also found that FGF21 was dispensable for the weight loss effects (57, 58). Thus, several studies demonstrate that restriction of individual amino acids is sufficient to increase circulating FGF21, replicating the effect of a low protein diet. However, the relative role of any specific amino acid in driving this effect remains unclear, as prior studies reach different conclusions regarding the importance of specific amino acids. These conflicting results call into question whether or not a single or combination of amino acids are the trigger for FGF21 release, and what role this increase in FGF21 plays in protein restriction, which requires further investigation. Instead, some have suggested that increased FGF21 represents a metabolic stress signal (61, 124).

The general control nonderepressible 2 (GCN2) to activating transcription factor 4 (ATF4) pathway is thought to be a link between hepatic stress due to amino acid deficiency and physiological response. Likewise, this has been a candidate mechanism for the induction of FGF21 in the liver during dietary protein restriction, and indeed, there is increased phosphorylation of eukaryotic initiation factor 2α (eIF2α), which is downstream to GCN2, with protein restriction (46). Studies have shown that either global (110) or liver-specific (125) knockout of GCN2 does diminish FGF21 production within 3 weeks on low-protein diet, but Laeger et al. found that serum FGF21 levels and hepatic mRNA levels of *Fgf21* return to normal after 12 weeks, suggesting a redundant or compensatory mechanism. ATF4 is thought to be the transcription factor of *Fgf21* important for its production in response to liver stress, due to the presence of multiple binding sites upstream of the gene in the liver (54, 126). However, ATF4 was not necessary for FGF21 induction in a model of sulfur amino acid restriction (127), calling its role into question, at least in the context of amino acid depletion. These studies demonstrate that there is still much to uncover about the mechanism of FGF21 production in response to hepatic stress.

#### 2.1.4 Ketogenic Diet

In addition to dietary protein restriction, ketogenic diets have been found to increase plasma FGF21 levels as well. Consumption of a ketogenic diet is a means to restrict carbohydrate intake and switches the primary energy source to ketones, which are a product of fatty acid metabolism. Thus, ketogenic diets are commonly utilized to interrogate nutrient metabolism. Mice that are fed a ketogenic diet typically lose weight and have chronically elevated circulating FGF21 levels (128). FGF21 likely plays an important role in the weight loss associated with ketogenic diets, as FGF21 KO mice gained weight and developed hepatic steatosis when fed a ketogenic diet (99). In a more recent study, liver-specific FGF21-null mice were found to have diminished energy expenditure and a weight loss response to a ketogenic diet, but glucose handling was not affected (62). Further studies suggest that FGF21 increases in ketogenic diet-fed mice due to the low protein content of these diets, and not in response to ketones per se, and is thus a response to protein restriction (46, 53). This is supported by the lack of an FGF21 response to ketogenic diets in humans (129, 130), which tend to contain sufficient dietary protein. These results collectively suggest that hepatic FGF21 contributes to weight loss in response to a ketogenic diet. In humans, however, ketogenic diets have not been associated with elevated FGF21 (31, 43, 45), so this effect may be rodent-specific.

#### 2.1.5 Simple Sugar Consumption

Mice fed a chow diet and given ad libitum access to water containing either glucose, fructose, or sucrose showed increased hepatic FGF21 mRNA expression and plasma FGF21 levels after 6 hours, an effect that was also observed in humans after 24 hours of a dextrose infusion (67). This FGF21 response was found to be dependent on ChREBP. In line with this, sugar intake is increased in global FGF21 KO mice as well as Hep-FGF21 KO mice and is suppressed with hepatic overexpression of FGF21, suggesting that the source of FGF21 is the liver. Direct signaling to the brain is implicated in this response (67), suggesting an important liver-brain axis involving FGF21 in the regulation of sugar intake. Recent work has determined that *Klb* is expressed in the ventromedial hypothalamus (VMH) and that these FGF21 responsive neurons signal to reduce sucrose consumption (131).

Similar effects have been observed in human cohorts. In one small group of healthy participants (n=10), the consumption of 75 g fructose induced a robust 3.4-fold increase in plasma FGF21 levels after 2 hours, an effect that was delayed and less pronounced following glucose consumption (70). In a similar Danish study, a small group of 9 men were recruited to undergo 3 days of consumption of a hypercaloric carbohydrate-rich diet (20). Plasma FGF21 levels increased by 800%, an effect that was not observed when unsaturated fats were similarly overconsumed. This was traced back to increased *de novo* lipogenesis and glucose production in the liver, and accompanied by decreased adipose tissue lipolysis, evidenced by reduced plasma fatty acid levels and lower phosphorylation of HSL (Ser^660^) (20). Thus, simple sugar consumption appears to increase hepatic FGF21 production in both rodents and human subjects.

#### 2.1.6 Endoplasmic Reticulum Stress

The endoplasmic reticulum (ER) is the major site of protein folding and maturation, as well as lipid synthesis. The accumulation of unfolded or misfolded proteins or perturbed lipid metabolism leads to ER stress, characterized by the unfolded protein response (UPR) (132). Disrupted ER homeostasis has been consistently linked with metabolic disease progression, primarily due to chronic nutritional metabolic stress that contributes to obesity, diabetes, and NAFLD. Emerging evidence suggests that such metabolic conditions associated with increased ER stress also exhibit increased endogenous FGF21. Cultured hepatocytes respond to ER stress signals, such as dithiothreitol and thapsigargin, by increasing FGF21 expression and secretion in an ATF4-dependent manner (133). Recently, Jiang et al. showed that hepatic FGF21 expression increased during the UPR as a compensatory mechanism to alleviate hepatic ER stress and steatosis (134). While a clearly defined mechanism by which FGF21 coordinates ER homeostasis remains to be elucidated, this and other studies suggest that FGF21 contributes to the resolution of ER stress (95, 134).

#### 2.1.7 Effector Organs for Hepatic FGF21

##### 2.1.7.1 Brain

While FGF21 is not readily expressed in the brain, it can easily cross the blood brain barrier to exert effects on the central nervous system (135, 136) and in fact many of the metabolic effects of FGF21 seem to primarily be mediated by the brain. In humans, FGF21 is detectable in cerebrospinal fluid, and positively correlates with plasma FGF21 levels as well as BMI and fat mass (137). FGFR1 is found throughout the brain, but KLB is only seen in a few discrete areas such as the suprachiasmatic nucleus (SCN) and the nucleus of the solitary tract (NTS) (138, 139). These regions are also key regulators of energy homeostasis and many studies in which FGF21 is administered directly into the brain *via* intracerebroventricular (ICV) cannula demonstrate that signaling in the brain can accomplish many of the actions attributed to FGF21 such as elevation of thermogenic genes in adipose tissue, sympathetic mediated increase in energy expenditure, and weight loss (6, 140, 141). Food intake, however, has been shown to be both increased (142) and decreased (140) in response to ICV-FGF21. Using laser capture microdissection and subsequent RT-PCR of distinct nuclei throughout the brain, researchers determined that *Klb* mRNA is primarily found in the SCN and NTS (139). This is also supported by cFos induction following ICV injection which indicated neural activation in the SCN and NTS, along with the paraventricular nucleus (PVN) and dorsomedial hypothalamus (DMH), which are likely downstream due to their lack of *Klb* expression (140). When *Klb* is knocked out in the hypothalamus using *Camk2a*-Cre mice, the effects of FGF21 overexpression and ketogenic diet were lost including more light-phase activity and elevated corticosterone and decreased adrenocorticotropic hormone (ACTH) in plasma (139). This effect was not observed in mice deficient in *Klb* specifically in the hindbrain (Phox2b-Cre mice), suggesting that FGF21 does not target the hindbrain (139). In a follow-up to this study, other effects of FGF21 such as weight loss, increased food intake, increased energy expenditure, and improved blood glucose were all attenuated by *Klb* deletion in the brain (6). Hill and colleagues then were the first show that the metabolic response to protein restriction was mediated by FGF21 signaling in the brain using a similar *Camk2a*-Cre mouse model (51). They went on to demonstrate that while low-protein diet fed animals show a preference for a high protein source of energy, this is also ablated by loss of central FGF21 signaling (51). Recent work by Flippo et al. demonstrates that *Klb* deletion in glutamatergic but not GABAergic neurons blocks most of the effects of FGF21 signaling during low protein feeding including weight loss, increased food intake, and WAT browning (the appearance of uncoupling protein-1 (UCP1)-positive cells within WAT), but not increased glucose uptake (143). Furthermore, the same group show with a *Klb*-Cre co-receptor mouse that *Klb* is expressed in many parts of the brain including the arcuate nucleus (ARC), VMH, and the PVN (131) from which *Klb* mRNA expression has not been previously observed (139). When *Klb* was knocked out only in VMH (glutamatergic) neurons, mice lost their decreased preference for sucrose due to low protein diet, but there was no effect on body weight or energy expenditure (131). In addition, an FGFR1 agonist reduced sweet preference and subsequently reduced sugar intake in human subjects, supporting the notion that FGF21 signaling is important for macronutrient preference (144). In sum, emerging research suggests that many of the physiological effects of FGF21, including body weight regulation and nutrient preference, are mediated by the brain.

##### 2.1.7.2 Adipose Tissue

Various elegant genetic mouse models have been very insightful for unraveling the complexities of liver-derived FGF21 signaling to adipose tissue by perturbing FGFR1 and KLB. The essential FGF21 receptor cofactor, KLB, is highly expressed in white and brown adipose tissue. When KLB^fl/fl^ mice are crossed with Adiponectin-Cre^Tg^ mice, the *Klb* gene is excised and subsequently not expressed in adiponectin-expressing cells such as white and brown adipocytes. Mice deficient in KLB specifically from adipocytes (Ad-KLB KO), including both white and brown, were refractory to the beneficial effects of rFGF21 on glucose metabolism when consuming either a chow or HFD (145), suggesting that adipocytes are important effector cells for the insulin sensitizing effects of liver-derived FGF21. This finding was supported by a previous study on a similar HFD (146), but contrasted with other studies that reported that FGF21 signaling to adipose tissue is dispensable for its insulin sensitizing effects (147, 148). Notably, one of these previous studies utilized a different driver of Cre-recombinase, AP2, which is notoriously non-specific for adipocytes, thus yielding potentially confounding results (147), and the other utilized a global KLB KO mouse strain (148). These previous Ad-KLB KO models were designed to disrupt KLB expression in both white and brown adipocytes. Taken one step further, disrupting KLB signaling only in brown and beige adipocytes by crossing KLB^fl/fl^ mice with UCP1-Cre^Tg^ also significantly impaired FGF21-mediated improvements in insulin sensitivity (145), indicating an important role for FGF21 signaling to BAT. An important question as to whether WAT and BAT contribute equally, or by different direct or indirect mechanisms, to FGF21-mediated improvements in glucose homeostasis remains to be determined.

A similar FGF21 signaling disruption strategy is to target FGFR1, the major FGF21 receptor expressed in adipose tissue. This strategy, again using the less specific AP2-Cre^Tg^ line, similarly suggests the importance of adipose tissue as the effector organ for the acute insulin sensitizing response to FGF21, as Ad-FGFR1-null mice were also refractory to the insulin-sensitizing effects of FGF21 (147). A second study utilizing similar FGFR1 conditional knock out mice (also derived from AP2-Cre^Tg^ mice) exhibited worsened hepatic steatosis due to increased adipose tissue lipolysis in response to extreme fasting (48 hours) (149). These results highlight the importance of FGF21 signaling to adipose tissue in the maintenance of insulin sensitivity, introduce the notion that FGF21 signaling to adipose tissue limits lipolysis, and suggest an important FGF21 signaling axis between the liver and adipose tissue.

There is some evidence that adipose tissue mediates the acute effects of rFGF21 to improve insulin sensitivity and glucose homeostasis, but not the chronic effects of pharmacological FGF21 on body weight and energy expenditure (145). WT DIO mice given rFGF21 chronically (delivered *via* osmotic minipumps for 2 weeks) lost significant body weight due to increased energy expenditure, an effect that was maintained in Ad-KLB KO mice (145), suggesting that the chronic effects of rFGF21 on body weight are not directly mediated through adipose tissue. It is important to point out that while this study suggests a differential role for adipose tissue in mediating the acute vs. chronic effects of rFGF21, whether a similar role for adipose tissue in mediating the endogenous effects of Hep-FGF21 effects is not known. Whether endogenous Hep-FGF21 exerts different acute or chronic effects is also not clear.

Some models used to study hepatic FGF21 have uncovered an important hepatic-adipose FGF21 axis that may occur in mice and possibly humans. In several studies using either extremely fasted mice (>10 hours) or mice given the β_3_-adrenergic receptor agonist CL-316,243, both of which induce or mimic increased sympathetic outflow to WAT, Hep-FGF21 exerts beneficial effects on BAT and promotes browning of WAT (23, 150, 151). However, humans fasted for as long as 10 days, a fasting stimulus that is sufficient to increase plasma FGF21 levels, exhibited *reduced* BAT activity assessed by fluorodeoxyglucose (FDG) activity (44). Although it is known that prolonged fasting decreases BAT activity to conserve energy (152), this further underscores the importance of Hep-FGF21 during fasting, and represents yet another example of the different FGF21 physiology between rodents and humans. These conflicting findings also support the notion that different stimulators of FGF21 secretion may promote different physiological effects; in other words, the context in which FGF21 is produced is critically important.

##### 2.1.7.3 Heart

Initial reports suggested that the heart was not a primary target for FGF21 due to low expression of the critical FGF21 co-receptor KLB (153). However, subsequent studies revealed that the heart does express relatively robust levels of FGFR1 in addition to KLB, and is also a source of FGF21 (14). FGF21 treatment has been shown to be protective against oxidative stress in cultured cardiomyocytes (86), and against cardiac hypertrophy and myocardial infarction in mice (154). In addition, mice globally deficient in FGF21 exhibit increased rates of cardiac hypertrophy and inflammation with reduced capacity for fat oxidation (89). FGF21 KO mice fed an obesogenic diet also exhibited worsened cardiomyopathy, evidenced by elevated oxidative stress, cardiomyocyte hypertrophy, and reduced capacity for fat oxidation and autophagy (155). In addition, alcoholic cardiomyophathy is exacerbated in mice deficient in FGF21, potentially due to elevated oxidative stress and mitochondrial dysfunction (156). This phenotype was replicated in humans, in which it was found that cardiac oxidative stress was elevated in hearts from subjects with alcoholism, which positively correlated with cardiac and plasma FGF21 levels (156). Collectively, these studies suggest an important protective effect of FGF21 against potentially adverse cardiac events, and point towards an emerging role for FGF21 to regulate autophagy in cardiac tissue. However, cardiac FGF21 expression has also been shown to increase in failing human hearts (86) and in patients undergoing acute myocardial infarction (87), and as such has been described as an independent risk factor for increased carotid artery intima-media thickness (157). Elevated circulating FGF21 was recently found to be an independent predictor of major adverse cardiovascular events (88); however, it is not clear what the source of this elevated FGF21 was (i.e. liver, heart, epicardial adipose tissue, or ectopic adipose tissue within or around the heart). Thus, much like the increased FGF21 levels observed in obesity, it is not yet clear whether increased cardiac FGF21 reflects a compensatory response to combat FGF21 resistance, or whether it could be detrimental when expressed from the heart.

##### 2.1.7.4 Liver

As a major FGF21-producing organ, the liver could also provide an autocrine source of FGF21 that could be physiologically meaningful. However, some studies suggest that autocrine FGF21 is not a major regulator of hepatic energy homeostasis due to predominant expression of FGFR4, which has low affinity for FGF21 (158, 159). While it is clear that hepatic FGF21 could impact liver physiology *via* its profound effects on lipid and glucose metabolism, it likely does so by indirect mechanisms due to the low expression of FGFR1, the major FGF21 receptor, in the liver.

### 2.2 Stimuli for White and Brown Adipocyte-Derived FGF21

While much has been reported regarding the signaling pathways induced by exogenous FGF21 on adipocytes [reviewed in (160)], less is known regarding signaling related to endogenous white, brown, or beige adipocyte-derived FGF21 (Ad-FGF21). Emerging evidence suggests that Ad-FGF21 is regulated by peroxisome proliferator-activated receptor gamma (PPARγ), in contrast to Hep-FGF21 which is regulated by PPARα (16, 17, 72). FGF21 expression increases progressively with 3T3-L1 and primary mouse and human white adipocyte differentiation, an effect that is dependent on PPARγ but not PPARα (28). FGF21 transcripts in epididymal WAT from leptin receptor-deficient *db/db* mice were upregulated by PPARγ agonists rosiglitazone and carboxylic acid (72), suggesting that adipose expansion is associated with increased FGF21 production. Circulating FGF21 was also increased dose-dependently by rosiglitazone in *db/db* mice (72). Further, rosiglitazone also increased FGF21 mRNA expression from WAT, but not liver (72). This is in agreement with another study that showed that rosiglitazone increases FGF21 expression and secretion from 3T3-L1 adipocytes (80). Collectively, work from several groups has shown that PPARγ agonists increase Ad-FGF21, which could contribute to circulating FGF21 levels, suggesting that FGF21 expression is driven by PPARγ in adipocytes.

Elegant studies suggest that under specific metabolic conditions (i.e. fasting), WAT expresses abundant levels of FGF21 mRNA and protein, but that Ad-FGF21 does not contribute to the circulating pool of FGF21. This has been shown in mice undergoing extreme fasting ≥24 hours, evidenced by the complete absence of plasma FGF21 in fasted Hep-FGF21 KO mice but not Ad-FGF21 KO mice (39). Importantly, the mice utilized in this study were long-term fasted (16-24 hours), which has been previously shown to be a potent stimulus for hepatic FGF21 expression (39). Indeed, the plasma FGF21 levels reported in this study, between 2,500-3,500 pg/mL (39), could be considered supraphysiological at >5-10x previously reported basal levels (33, 41, 161, 162). Moreover, and in contrast to Hep-FGF21, Ad-FGF21 deficiency did not alter glucose or insulin metabolism (39). While neither Hep-FGF21 KO or Ad-FGF21 KO impacted body weight in mice in this study, body fat mass tended to be lower in the absence of Ad-FGF21 (39). Thus, while evidence suggests that Ad-FGF21 is induced by various stimuli that differ considerably to those for Hep-FGF21 (to be discussed in the following sections), Ad-FGF21 may not circulate based on particularly nuanced studies to date. However, as described in this and later sections, particular physiological stimuli can render brown and/or beige adipocytes, and potentially white adipocytes, as a source of circulating FGF21. Thus, FGF21 could potentially be considered an adipokine and batokine (163).

#### 2.2.1 Overfeeding and Obesity

As discussed above, conditions associated with overfeeding, including obesity, are also associated with increased systemic FGF21 levels. This has been shown consistently in humans and rodents fed a HFD, diets high in carbohydrates, and ketogenic diets (3, 20, 72, 73, 128), as well as humans with obesity (26–28, 76) and in humans that have been acutely overfed (164). In many of these studies, mRNA and protein expression of FGF21 have consistently been shown to be increased in WAT in the setting of overnutrition (29, 39, 72), raising the possibility that Ad-FGF21 could be a source of circulating FGF21 in this context. While several studies have shown that Ad-FGF21 does not circulate (39, 79, 165), it is important to note that the particular nutritional settings under which these previous studies were performed (i.e. extreme fasting or adherence to a very-low calorie diet) may have masked any contribution to circulating FGF21 from adipose tissue. Thus, while overnutrition increases circulating FGF21 levels and adipose tissue FGF21 mRNA expression, it has not been conclusively demonstrated that circulating FGF21 derives from adipose tissue in that context.

#### 2.2.2 Genetic Models of Obesity

Mice deficient in the leptin gene, termed *ob/ob* mice, develop spontaneous obesity due to dysregulated hyperphagia when fed a chow diet, and as such are a common model of genetic obesity. An initial study by Lundasen et al. showed that hepatic *Fgf21* mRNA expression increased by 12-fold in ob/ob mice relative to lean controls (18). Another study by Badman et al. reported that genetically obese *ob/ob* mice exhibit increased circulating FGF21 levels, with concomitant increased *Fgf21* expression from the liver and WAT (75). A later study by Hale et al. confirmed that *ob/ob* mice exhibit even higher plasma FGF21 levels than mice fed a HFD (35). While WAT *Fgf21* mRNA levels were increased in ob/ob mice relative to chow-fed C57Bl/6J controls, *Fgf21* mRNA was increased a staggering 50-fold in the liver (35, 75). While is seems prudent to assume that circulating FGF21 derived from the liver in these *ob/ob* mice, the nutritional status of the animals at the time of blood and tissue collection was not apparent (i.e. whether the mice had been fasted prior to sacrifice), so it is difficult to speculate on the tissue origin of FGF21 in this model. Another similar model of genetic obesity is the *db/db* mouse, which is characterized by deficiency of the leptin receptor, and leads to spontaneous obesity *via* a similar disruption in leptin signaling. FGF21 protein from subcutaneous and visceral WAT has been shown to be higher in obese *db/db* mice compared with lean littermates (28), suggesting an important role for Ad-FGF21 in genetic obesity models in addition to the better characterized Hep-FGF21.

#### 2.2.3 Cold Exposure

For some time evidence has supported a model where FGF21 was secreted from adipose tissue in response to cold exposure and that this signal was necessary for increased UCP1-dependent increases in energy expenditure. Cold exposure has been shown to induce *Fgf21* transcript expression and protein release in BAT of mice, and cultured brown adipocytes produced FGF21 when treated with norepinephrine, but FGF21 levels in circulation are not altered by cold exposure (22). Cold-induced BAT *Fgf21* mRNA expression was confirmed in an additional study, which also showed that increased systemic FGF21 levels were BAT-derived (23), an effect attributed to long term cold exposure (>24 hours). Subsequent studies in mice and rats support the potential for BAT-derived FGF21 to circulate (23, 24, 82). Moreover, accumulating evidence suggests that Hep-FGF21 mRNA expression levels *decrease* with cold exposure, and are thus not likely to contribute to cold-induced increases in circulating FGF21 (23, 24, 79). Data in humans has also supported this with FGF21 measured in blood elevated with a mild cold exposure of 19°C (83). One study showed that *Fgf21*-KO animals have a blunted response to cold exposure and had a lower body temperature and thermogenic gene response in adipose tissue, however, energy expenditure was not measured (79). However, another study demonstrated that FGF21 from the liver and not from adipose tissue was important for a cold exposure of three days and that its effects were mediated by KLB receptors in the brain not adipose tissue (74). Increasingly, evidence now suggests that the increase in energy expenditure observed with FGF21 signaling is due to the hormone secreted from the liver and is centrally mediated (51, 141, 143), and that the response to adaptive cold exposure is FGF21-independent. In a study of protein restriction-induced FGF21, *Ucp1*-deficient mice do not increase energy expenditure in response to low-protein diet, but FGF21-deficient mice respond normally to cold exposure (47). In a double knockout of UCP1 and FGF21, animals were still able to maintain increased thermogenesis during an extended cold exposure of 3 weeks, contradicting the previous study of FGF21 KO mice (166). Recently, a study was not able replicate an increase in energy expenditure when animals were administered FGF21 *via* osmotic minipump, and suggested that FGF21 increases the body temperature set point and accomplishes this by reducing heat loss, not UCP1 and adipose tissue-mediated increased energy expenditure, possibly introducing another method by which FGF21 can influence body temperature (167). Thus, while it is likely that BAT expresses FGF21 in response to cold, and that BAT-derived FGF21 may circulate under particular cold conditions, a clear physiological role for cold-induced FGF21 has recently come into question. There is now speculation that FGF21 may play a role in the nutrient mobilization required during cold exposure (168). We cannot exclude the possibility that BAT-derived FGF21 plays a direct role in adaptive thermogenesis.

It is important to point out that while cold exposure and sympathomimetics induce Ad-FGF21 gene and protein expression from WAT and BAT in both rodent models and humans, this effect from WAT is much more pronounced in rodents than in humans (169). Human WAT expresses negligible amounts of FGF21 mRNA, while human perirenal BAT displays a robust FGF21 expression pattern in pheochromocytoma patients and healthy controls (169). FGF21 gene expression positively correlated with UCP1 expression, indicating that human BAT expresses detectable FGF21. Similarly, healthy men with detectable BAT exhibit higher plasma levels of FGF21 than those with undetectable BAT (170), suggesting that BAT is a source of circulating FGF21 in cold-exposed humans. By contrast, rodents have consistently shown robust FGF21 mRNA expression patterns from both WAT and BAT (3, 24). This important species difference in FGF21 expression between WAT and BAT depots should be considered when assessing the potential translational implications of Ad-FGF21 kinetics.

#### 2.2.4 Adipocyte-Derived FGF21 Signaling to Effector Tissues

While both white and brown adipocytes express abundant levels of FGF21, most research to date suggests that Ad-FGF21 behaves in an autocrine fashion and may not circulate in measurable levels (39, 79, 171). However, as described in more detail below, the concept that following particular metabolic or physical stressors Ad-FGF21 can circulate (most notably from BAT) is gaining traction. Importantly, as mentioned in previous sections, research into endogenous FGF21 has consistently revolved around a hepatocentric viewpoint. The liver without question is the most dynamic source of systemic FGF21; however, the attention given to Ad-FGF21 has been limited at best. Due to particular experimental models utilized in most studies of Ad-FGF21, including extreme fasting, the physiological role of Ad-FGF21 may often be masked and subsequently overlooked.

Conditional knock out mice that perturb the FGF21 signaling pathway have contributed to the dogma that adipocytes secrete FGF21 in an autocrine manner. Ad-KLB KO mice fed a HFD for 12 weeks did not exhibit a body weight phenotype in comparison with WT mice (136, 145), suggesting that either (1) liver-derived FGF21 does not mediate body weight *via* effects on adipose tissue, or (2) autocrine adipocyte FGF21 signaling does not influence body weight. Hep-FGF21 KO mice displayed equivalent WAT browning as wild-type mice in response to CL-316,243, a β_3_-adrenergic agonist utilized to mimic increased sympathetic outflow (150). Similarly, Ad-KLB KO mice were refractory to the effects of CL-316,243 on WAT browning (150). Collectively, these studies provide evidence that Ad-FGF21 signals in an autocrine manner in response to 12 weeks of HFD-feeding or a sympathomimetic. Whether Ad-FGF21 signals in an autocrine fashion in response to other stimuli remains to be conclusively determined.

Recent work by Abu-Odeh et al. suggests that Ad-FGF21 signaling pathways may differ from Hep-FGF21. Primary white adipocytes responded to the sympathomimetic CL-316,243 by increasing FGF21 expression and secretion that was dependent on the p38/PKA pathway, but independent on the well-described ERK1/2 pathway (150). This is in stark contrast to previous work showing that ERK1/2 activation is required for FGF21 signaling (14, 141, 172). The authors speculate that the mechanism of thermogenic activation of FGF21 (via PLC) may differ from other stimuli.

A recent study by Kusminski et al. showed that dysfunctional white adipocytes, engineered in this case to overexpress the mitochondrial protein ferritin, exhibited increased *Fgf21* mRNA expression (38), to levels similar to what is observed in obesity. Importantly, these dysfunctional adipocytes were found to contribute to circulating FGF21 levels (38), reaching concentrations consistent with what has been observed in mouse models of obesity (3). Because dysfunctional adipocytes are also observed in the obese state, it is therefore plausible that Ad-FGF21 could contribute to the elevated circulating FGF21 observed in models of obesity. Moreover, several studies have shown convincing correlations between adipose FGF21 mRNA expression and circulating FGF21 levels in both humans and rodent models of obesity (3, 28, 77). Thus, additional studies are required to tease apart the contribution of white Ad-FGF21 to circulating FGF21 levels that are characteristic of obesity, and whether Ad-FGF21 can impact other organs systemically, such as the brain.

The concept that BAT can secrete bioactive endocrine substances that regulate energy metabolism is emerging (163). Based on elegant knock out mouse models and fat transplantation experiments, FGF21 could potentially be classified as such a batokine. Keipert et al. showed that mice globally deficient in UCP1 expressed high levels of FGF21 mRNA from WAT and BAT with progressively severe cold exposure (24), although only FGF21 derived from BAT was found to circulate. Hepatic and skeletal muscle tissue did not display increased FGF21 mRNA levels, suggesting that the increase in circulating FGF21 derived from BAT (24). Similarly, mice that received BAT transplantation exhibited increased circulating FGF21 levels that were found to be BAT-derived, with no changes in Hep-FGF21 gene expression (173). An elegant study by Ruan et al. showed that BAT-derived FGF21 likely targets the heart in an endocrine manner, where it plays an important role in hypertrophic cardiac tissue remodeling. Collectively, these studies suggest that FGF21 could be considered a batokine under particular physiological conditions, with the potential to signal to other tissues.

### 2.3 Stimuli for Pancreatic FGF21 Production

The pancreas is a major FGF21-expressing organ shown in some studies to express higher levels of FGF21 mRNA and protein than the liver and WAT (21, 81, 84), although the precise function of pancreatic FGF21 remains an enigma. The majority of FGF21 protein appears to derive from acinar tissue, with some also observed in islets (21). Interestingly, unlike the liver, pancreatic FGF21 expression *decreases* following fasting (81, 84), suggesting that pancreatic FGF21 regulation is distinct from the liver. Similarly, administration of recombinant FGF21 appears to down-regulate FGF21 expression in the pancreas (21). While there are a very limited number of studies that illuminate pancreatic FGF21 function, a few point towards a beneficial effect of pancreatic FGF21 on glucose metabolism. Young FGF21 KO mice do not exhibit a distinct phenotype in the pancreas, but exhibit higher body weights and glucose intolerance (21). However, aging increased islet surface area, and older FGF21 KO mice challenged with a HFD developed exacerbated islet hyperplasia (21). FGF21 KO mice fed a HFD for 16 weeks (started at 24 weeks of age) weighed the same as WT mice (21). As the authors did not present weight curves, it is not possible to determine whether weight gain was similar between the genotypes. In a similar study, mice globally deficient in FGF21 exhibited normoglycemia, but impaired glucose and insulin tolerance due to dysregulation of insulin secretion by islets (174). In addition, FGF21 appears to be required to protect mice against experimental pancreatitis, as FGF21 KO mice exhibit more damage, while FGF21^Tg/Tg^ mice are protected from damage, following cerulein-induced pancreatitis (85). Additionally, mice globally deficient in FGF21 are more susceptible to pancreatic ER stress than WT mice, an effect that is reversed by rFGF21 administration (84). Unlike in other FGF21-secreting tissues, FGF21 in the acinar pancreas appears to stimulate PLC-IP_3_R signaling to increase intracellular calcium, leading to phosphorylation of ERK1/2 (84). In contrast to the liver, pancreatic FGF21 likely serves an autocrine/paracrine function (84). In summary, while relatively little is known about pancreatic FGF21, it may serve to alleviate pancreatic stress and thus contribute to glucose homeostasis. There is also evidence that pancreatic FGF21 signals *via* different pathways than in other tissues.

### 2.4 Stimuli for Skeletal Muscle FGF21 Expression and Secretion

Under basal conditions, skeletal muscle is not a significant source of FGF21 (153). However, conditions promoting muscle stress have been reported to increase FGF21 expression from skeletal muscle (SM-FGF21). FGF21 protein expression from skeletal muscle in response to fasting (48 hours) has been reported to be comparable to liver expression levels in one study (93), then shown to be unchanged in another (24 hours of fasting) (175). FGF21 mRNA and protein expression and secretion can be induced by insulin in mice and humans (93, 94) and by transgenic AKT overexpression from skeletal muscle cells in culture (93). Transgenic mice overexpressing AKT from type Ilb skeletal muscle fibers exhibited an increase in circulating FGF21 levels (93), suggesting that SM-FGF21 is regulated by AKT, and can circulate. Healthy young men infused with insulin exhibit increased SM-FGF21 mRNA expression that mirrors increased plasma levels, and hyperinsulinemic men also had elevated skeletal muscle FGF21 mRNA expression (94). These initial findings raise many questions about the regulation of SM-FGF21, as insulin levels are low in the fasting state, yet both fasting and insulin appear to induce SM-FGF21. Based on published work to date, the consensus appears to be that insulin is the more robust stimulus for SM-FGF21 expression, rendering it akin to Ad-FGF21. This again supports the notion that different signaling pathways are involved in the expression and secretion of FGF21 from different tissues.

Some evidence suggests that SM-FGF21 exerts a protective effect in response to cellular stress. Mice engineered to overexpress UCP1 ectopically from skeletal muscle displayed a surprising increase in SM-FGF21 mRNA expression and a 5-fold increase in circulating FGF21 levels (176). Interestingly, this increased SM-FGF21 led to the browning of WAT (176), suggesting a novel skeletal muscle-adipose FGF21 axis. Additional work showed that mice devoid of skeletal muscle mitochondrial autophagy capacity (SM-ATG7 KO mice, a model of mitochondrial dysfunction) exhibited increased SM-FGF21 that circulated (95). SM-ATG7 KO mice also exhibited resistance to diet-induced obesity and browning of WAT, for which SM-FGF21 was indispensable (95). Similarly, a study in which mitochondrial respiratory chain deficiency was engineered in mice resulted in increased SM-FGF21 levels that evoked resistance to diet-induced obesity and associated dyslipidemia and hepatic steatosis (175). In one study by Ribas et al., SM-FGF21 was shown to be expressed in fully differentiated myoblasts and in response to mitochondrial stress, requiring MyoD and ATF2 (177). In a different study by Kim et al., SM-FGF21 was found to be regulated by ATF4 (95), a master regulator of the integrated stress response, highlighting the potential pleiotropic effects of FGF21 due to its involvement with different signaling pathways. Indeed, human subjects with muscle-manifesting mitochondrial respiratory chain deficiencies have been shown to have increased serum FGF21 levels (97). Further, mice with conditional transgenic overexpression of perilipin 5 (PLIN5) from skeletal muscle displayed increased FGF21 expression which reached the circulation and caused WAT browning (96). These findings again suggest that under particular conditions, non-hepatic FGF21 can circulate, and SM-FGF21 could be classified as a “myokine”. There is also some evidence to suggest a potential autocrine role for FGF21 in skeletal muscle, with a potentially important role for SM-FGF21 on myofiber type development (178). Thus, the metabolic regulation and signaling pathways governing SM-FGF21 are quite complex and require further study.

Aerobic exercise is a well-known stimulant for FGF21 (179). Acute and chronic aerobic exercise has been associated with increased circulating FGF21 levels in many studies in humans (25, 90–92, 180–182). The tissue source of FGF21 was not investigated in these studies, and was assumed to be liver-derived. While the majority of exercise-induced FGF21 is thought to derive from the liver, emerging evidence suggest that skeletal muscle may also contribute to the circulating pool. Mice conditioned to running wheels (15-30 m/min, 3x/week) exhibited increased SM-FGF21 mRNA and protein expression, as well as increased Hep-FGF21 expression and plasma FGF21 levels (25). Notably, these mice were overnight fasted prior to tissue and blood collection, thus potentially masking an acute contribution of exercise to SM-FGF21. Further work is therefore warranted to determine if SM-FGF21 contributes to the increased systemic FGF21 levels observed following exercise.

### 2.5 Summary of Endogenous FGF21 Stimuli

FGF21 is an extraordinarily complex protein that is secreted by many different tissues in response to a myriad of environmental, nutritional, and metabolic stimuli (see **Table 1**). Over a decade of elegant studies have contributed to our knowledge regarding the complicated signaling and metabolic effects of endogenous FGF21. The majority of attention has been given to Hep-FGF21, contributing to the notion that the liver is the primary source of FGF21 under most metabolic conditions, including fasting, protein restriction, alcohol and simple sugar intake. However, the contributions of other tissues to FGF21 signaling and metabolic function could be important under particular metabolic conditions, such as cold exposure, obesity, or during exercise. What makes the existing literature surrounding FGF21 so complex and difficult to interpret is that the FGF21 response by the liver, adipose, skeletal muscle, and pancreas varies so greatly by the nature of the metabolic stimulus. This renders the particular methodology under which FGF21 effects are studied exquisitely important for the complete interpretation of phenotypes, and we stress that dogma attributed to tissue-specific effects of FGF21 should be used with caution.

## 3 Pathological Conditions Associated With FGF21

### 3.1 FGF21, NAFLD, and NASH

Somewhat paradoxically, elevated plasma FGF21 levels are observed in patients with NASH (32, 183–190), with similar trends observed in animal studies (191, 192). Plasma FGF21 levels in mice with NAFLD positively correlate with hepatic fat content (193). NAFLD has also been reported to reduce hepatic KLB, FGFR2, and FGFR4 expression, suggesting reduced autocrine FGF21 signaling in the liver (194). This implied FGF21 resistance could be driven by increased inflammatory cytokines that are frequently observed in NAFLD (195). Interestingly, some research suggests that elevated FGF21 levels observed in patients with NAFLD could be independent of PPARα signaling in the liver (193), in contrast to what we understand about fasting-induced hepatic FGF21. Conversely, a recent study has shown that fenofibrate, a potent PPARα agonist, increases hepatic FGF21 expression and plasma FGF21 levels (196). In concert, fenofibrates also reduce HFD-induced body weight gain by dramatically reducing WAT mass and by increasing the browning of WAT (196). Mice deficient in FGF21 did not respond to fenofibrates with browning, suggesting an important signaling axis between the liver and WAT that requires FGF21. While there is a robust association between FGF21 and NAFLD, whether FGF21 is simply a biomarker or intricately involved in the progression of NAFLD is not yet known.

### 3.2 Obesity and FGF21 Resistance

#### 3.2.1 Human Obesity

Serum FGF21 was measured from 232 Chinese men and women, mean age 55 years (28). No differences were observed between men and women, but people with overweight/obesity (mean BMI 28.6) had serum FGF21 levels of 291.8 ng/L (range 144.5-512.0), while lean controls (mean BMI 22.3) had significantly lower FGF21 levels of 208.7 ng/L (range 94.4-325.7) (28). Serum FGF21 levels positively correlated with adiposity, body mass index (BMI), waist circumference, waist-hip ratio, fat percentage, homeostatic model assessment for insulin resistance (HOMA-IR), triglycerides, and serum adipocyte fatty acid binding protein (A-FABP) levels, and negatively associated with serum adiponectin and high density lipoprotein (HDL) cholesterol levels (28). Serum FGF21 levels were also significantly elevated in subjects with metabolic syndrome (defined as having 3 or more of the following criteria: central obesity (waist circumference ≥ 80 cm in women and 90 cm in men), hypertriglyceridemia (fasting triglycerides ≥ 1/69 mmol/L), low HDL cholesterol (fasting HDL < 1.29 mmol/L in women and < 1.04 mmol/L in men), hyperglycemia (fasting glucose ≥ 5.6 mmol/L or taking oral hypoglycemic agents for the treatment of type 2 diabetes), or hypertension (sitting blood pressure ≥ 130/85 mmHg) (28). Plasma FGF21 levels were also measured in a separate cohort of men and women in Mexico (n=241), and also showed a positive correlation between serum FGF21 levels and body weight and waist circumference (76). Similarly, serum FGF21 positively correlated with BMI, waist circumference, and adiposity in healthy men and those with diabetes in Tanzania (77). Collectively, these data suggest that FGF21 levels rise during obesity in humans, and could derive from adipose tissue.

In one recent study, 90 young gender-matched individuals were stratified as having normal weight (n=30) or classified as having overweight or obesity (n=60). The overweight individuals were further classified as insulin sensitive with overweight or obesity (ISO, HOMA-IR<2.5) or insulin resistant with overweight or obesity (IRO, HOMA-IR≥2.5) (n=30 each) (3). Subjects with ISO had significantly higher subcutaneous fat area and lower visceral fat than individuals with IRO (3), consistent with previous studies (197, 198). Both overweight groups had elevated FGF21 levels compared with lean controls, which positively correlated with subcutaneous fat area, but not visceral fat area (3). These data suggest that subcutaneous fat may contribute to circulating FGF21 levels in obesity, and may not impact insulin resistance.

#### 3.2.2 Mouse Models of Obesity

Several studies have reported elevated circulating FGF21 levels in rodent models of obesity. Initial studies reported increased serum FGF21 levels in genetically obese *ob/ob* (35, 75) and *db/db* (28) mice. In an early study, C57Bl6 mice that were fed a high fat high sucrose diet for 22 weeks exhibited a 20-fold and 2-fold increase in liver and perigonadal FGF21 mRNA expression, respectively, coupled with a 3-fold increase in plasma FGF21 levels (33). A pivotal study by Li et al. introduced the notion that FGF21 is a critical player in the expansion of subcutaneous fat required for obesity. FGF21 KO mice reportedly gained less body weight and subcutaneous body fat on a HFD (16 weeks, 45% fat) (3). Replenishing FGF21 KO mice with obesity-mimicking levels of recombinant FGF21 for 4 weeks did not alter body weight, but restored subcutaneous fat mass after 8 weeks of HFD, suggesting that FGF21 is important for subcutaneous fat expansion in obesity. Mice that were deficient in KLB specifically from adipocytes were refractory to the effects of recombinant FGF21 to increase subcutaneous fat mass (3), suggesting that direct FGF21 signaling to adipocytes is required for subcutaneous fat expansion in obesity. Deficiency of FGF21 caused a phenotype characterized by reduced adipogenesis and insulin responsiveness, with notable reductions in *Cebpa*, *Srebf1a*, *InsR*, *Irs1*, *PI3k*, and *Glut4* mRNA from subcutaneous fat that was restored by recombinant FGF21 injection. These effects were replicated *in vitro*, in which primary white adipocyte differentiation from subcutaneous precursors was delayed in the absence of FGF21 expression (3).

Similarly to the study by Li et al., mice with global KLB deficiency were slightly leaner than their WT counterparts at 10 weeks of age (199), an effect that had been previously reported (200, 201). KLB KO mice fed a HFD were much leaner and gained much less body weight, reflected by reduced lean and fat mass, with increased energy expenditure. WAT from KLB^-/-^ mice exhibited increased expression of lipolysis genes (ATGL, HSL, and lipoprotein lipase (LPL)), decreased inflammatory genes (tumor necrosis factor (TNF) and interleukin-6 (IL6)), and increased adiponectin. UCP1 was decreased in WAT, excluding browning as a mechanism for fat loss. However, UCP1, PGC1a, and type II iodothyronine deiodinase (DIO2) were increased in BAT, indicative of increased BAT-mediated thermogenesis. In addition, glucose tolerance was improved in high fat-fed KLB KO mice. Interestingly, hepatic FGF21 expression was increased in KLB KO mice. The expected outcome of global KLB deficiency was worsened metabolism (i.e. hepatosteatosis, obesity, insulin resistance) under HFD feeding conditions. Because the opposite phenotype was observed, this raises questions about the role of endogenous FGF21 signaling in different metabolic conditions.

Adding to the complex relationship between obesity and increased FGF21, it has been recently shown that UCP1-KO mice, which are resistant to diet-induced obesity at ambient 23⁰C, displayed a 2.5-fold increase in serum FGF21 levels when fed a HFD (202). This rise in FGF21 likely originated from WAT and BAT, where mRNA expression levels were robustly increased, with no change observed in the liver. The authors speculated that BAT-derived FGF21 could signal to inguinal WAT to promote browning (202), which could have profound metabolic impacts related to obesity resistance.

#### 3.2.3 FGF21 and Weight Loss

In addition to evidence that FGF21 levels increase with excess adiposity, there is now mounting evidence that energy restriction decreases FGF21 levels in rodents and humans. A recent weight loss intervention study recruited 195 subjects (including 181 men and women with obesity and 14 normal-weight controls) (26). Participants with obesity had ~6-fold higher serum FGF21 levels than lean controls at baseline, an effect that was equivalent in men and women (26). The subjects with obesity underwent weight loss by either following a very low calorie ketogenic diet (VLCKD), a low calorie diet, or bariatric surgery, with 4-6 months of follow-up. VLCK- and LC-mediated weight loss were both accompanied by a reduction in serum FGF21 levels that mirrored the amount of fat lost. Subjects that then regained some of the weight lost experienced significant increases in FGF21 levels. By contrast, subjects that underwent bariatric surgery (two separate cohorts that underwent Roux-en-Y gastric bypass, biliopancreatic diversion, or sleeve gastrectomy), which resulted in a similar total degree of weight loss as the VLCKD group, experienced an *increase* in serum FGF21 levels. The authors speculate that while the final degree of weight loss achieved was similar between the two weight loss regimens, the rate of weight loss was initially much faster in the bariatric surgery group, which also achieved a lower degree of reversal of insulin resistance, both of which could drive differential FGF21 secretion rates (26). The divergence of FGF21 kinetics in low-calorie weight loss vs. bariatric surgery has been reported previously (203). Several other studies report that FGF21 levels decrease in response to lifestyle weight loss interventions (204, 205). However, another study showed no change in plasma FGF21 levels following a low calorie diet, but subsequently showed that arterial and subcutaneous WAT-derived FGF21 levels increased postprandially following a single high fat meal (165). In stark contrast with the general consensus that weight loss due to lifestyle interventions decreases FGF21 levels, weight loss due to bariatric surgery has been reported to *increase* FGF21 (203, 205–207). One study reported an acute postprandial increase in FGF21 levels only in patients who had undergone bariatric surgery (n=14) compared with control subjects experiencing similar negative energy balance (208). While FGF21 levels appear to increase more acutely following bariatric surgery, longitudinal data suggest this effect is transient, with FGF21 levels reported as being unchanged 12 months after surgery (205, 209, 210). It is possible, as we suspect that FGF21 levels are induced in response to metabolic stress, that prolonged fasting, obesity, and the severe muscle loss induced by bariatric surgery could all increase circulating FGF21 levels (43, 44). We have previously shown that FGF21 is dispensable for the dramatic weight loss and metabolic improvements observed following bariatric surgery, as mice globally deficient in FGF21 exhibited equivalent weight loss and body compositional changes as control mice following bariatric surgery (211). Thus, a discernable role for FGF21 in bariatric surgery-mediated weight loss also remains elusive.

#### 3.2.4 FGF21 Resistance in Obesity

Initial studies that observed increased FGF21 levels associated with obesity hypothesized that this reflected a state of “FGF21 resistance”, due to modestly decreased expression levels of both KLB and FGFR1 in perigonadal WAT (33). In addition, obese mice injected with low levels of rFGF21 exhibited reduced liver and WAT phospho-ERK1/2 levels, suggesting reduced FGF21-related signaling (33). However, whether FGF21 resistance exists, and whether it explains the obesity-associated rise in circulating FGF21 levels is still under debate. First, *ob/ob* and diet-induced obese mice were shown to remain responsive to rFGF21 in later studies, evidenced by hepatic and WAT ERK phosphorylation levels that were equivalent to lean controls in response to increasing doses of rFGF21 (35), and at high rFGF21 doses in the initial study (33). Second, mice that are deficient in KLB specifically in WAT are also still responsive to the effects of rFGF21 (145). Third, mice with transgenic KLB overexpression are not protected from diet-induced obesity (36), despite the prediction that they would be more FGF21-sensitive. Finally, initial reports of decreased FGF21 receptor and co-receptor expression in the context of obesity have been challenged by additional studies that show no change or even increased expression of these receptors in adipose tissue from human subjects with obesity (212, 213).

The first study published in opposition to the FGF21 resistance hypothesis showed that while *ob/ob* mice have much higher FGF21 levels than diet-induced obese mice, which one would predict would make them more FGF21 resistant, they did not display reduced phospho-ERK or other overt signs of FGF21 resistance (35). Second, the marked reduction in FGFRs and KLB did not result in reduced phospho-ERK. Third, diet-induced obese and *ob/ob* mice exhibited equivalent metabolic responses to rFGF21. Resistance to other endocrine hormones, such as leptin and insulin, require a higher exposure to these hormones to achieve their target effects (i.e. to regulate food intake and glucose control). Because the same dose of rFGF21 produced similar metabolic effects in DIO and *ob/ob* animals, it is unlikely that FGF21 resistance had occurred. The discrepancies between this study and the initial study that suggested that obesity is an FGF21-resistant state (33) could potentially be explained by different functional readouts and doses of rFGF21 utilized.

Another study to challenge this hypothesis showed that while KLB gene and protein expression were reduced in epididymal WAT from diet-induced obese mice, KLB expression levels were unchanged in liver and BAT (36). The authors hypothesized that overexpressing KLB from white adipocytes would reduce obesity-associated FGF21 levels, if indeed they were reflective of FGF21 resistance. Transgenic mice overexpressing FGF21 specifically from adipocytes (Adipo-KLB^Tg/Tg^) exhibited no overt phenotype when challenged with a HFD, and expressed comparable levels of FGF21 in liver and plasma as WT mice (37). The authors interpret this as suggestive that adipocyte-derived KLB has no effect on obesity-mediated FGF21 resistance, but it could also be that adipocyte-KLB is not required for obesity-associated FGF21 increases. Collectively, these data highlight the ambiguity over the concept that obesity is an FGF21-resistant state, and suggest that there may be as yet unknown pathways by which adipocytes respond to FGF21.

### 3.3 FGF21 Associations With Muscular Diseases

As briefly mentioned in Section 2.4, skeletal SM-FGF21 is associated with some pathological conditions. Human subjects with muscle-manifesting mitochondrial disorders have been shown to have increased serum FGF21 levels (97). Adults and children were included who had been diagnosed with various skeletal and/or cardiac muscle-affected mitochondrial disorders, including (but not limited to) cardiomyopathy, T2DM, myalgia, mitochondrial neurogastrointestinal encephalomyopathy (MNGIE), mitochondrial recessive ataxia syndrome (MIRAS), or myotonic dystrophy type 2. Subjects with confirmed mitochondrial disorders had on average 10-fold elevated serum FGF21 levels than healthy control subjects, which was positively associated with severity of symptoms (97). Subjects with disorders that do not primarily impact skeletal muscle, such as MIRAS (which primarily affects the nervous system) or hepatic dysfunction, had the lowest levels of FGF21, suggesting a specific association with skeletal muscle pathologies (97). This was supported by elevated FGF21 mRNA expression in a small cohort of subjects that positively associated with serum levels. These findings are supported by a previous study reporting that mouse models of respiratory complex disruption also exhibit increased SM-FGF21 mRNA expression and increased serum FGF21 levels (175).

Similarly, FGF21 may also be a biomarker for cardiovascular disease. Several studies to date have suggested a positive association between coronary artery disease and serum FGF21 levels (214–216). Moreover, serum FGF21 levels have been shown to predict major cardiac adverse events in humans (88), including non-fatal myocardial infarction, non-fatal stroke, hospitalization due to angina pectoris, and cardiac death. Indeed, FGF21 has been shown to be expressed in the heart as part of the stress response to cardiac hypertrophy, cardiac remodeling, and myocardial infarction (89, 217). Thus, cardiac diseases are also associated with elevated FGF21 levels that may derive from the heart itself.

### 3.4 Potential for FGF21 Gene Editing as a Treatment For Metabolic Disease

Pharmacological FGF21 has been investigated for nearly 15 years as a potential therapeutic against obesity, T2DM, and NAFLD (reviewed in [8, 9, and (10)]. Because the native FGF21 protein has poor pharmacokinetic properties, various FGF21 analogs and mimetics have been developed to evade proteolytic cleavage, aggregation, and rapid clearance (218, 219). However, despite marginal improvements in efficacy and half-life, current FGF21 mimetics require frequent administration and promote uncomfortable side effects in patients. Thus, there is potential for gene editing to increase endogenous production of FGF21 for metabolic benefit. Jimenez et al. recently showed that overexpressing FGF21 from various tissues, including the liver and adipose tissue, rendered mice resistant to high fat diet-induced obesity and associated tissue inflammation and insulin resistance (220), essentially phenocopying previous FGF21-transgenic mouse studies (4, 102, 221). Although the study designs differed considerably between the hepatic- and adipose-AAV approaches, it seemed that the metabolic effects of overexpressing FGF21 from the liver were more pronounced than from adipose tissue (220). While the study by Jimenez et al. showed robust induction of circulating FGF21 levels with this AAV approach, and that such induction improved obesity and its associated comorbidities, they did not truly isolate tissue-specific effects of FGF21 expression. It would be interesting to repeat their experiments using mice globally deficient in FGF21 to determine whether gene editing from a particular tissue yielded different metabolic effects.

## 4 Concluding Remarks

With more than a decade of research into the physiological effects of FGF21 on energy metabolism, several gaps in our knowledge base remain. For one, the emerging and extremely varied tissue-specific stimuli for FGF21 expression introduce many questions. Why would such diametrically opposed stimuli (i.e. fasting vs. overfeeding) both trigger FGF21 expression from different tissues? Could the tissue source of FGF21 differentially dictate its functionality due to unique features of secretion dynamics, tissue location, extracellular microenvironment, or posttranslational modification? Further, it is still not clear why FGF21 administration tends to improve metabolic endpoints, yet FGF21 levels are paradoxically increased with metabolic conditions such as obesity, type 2 diabetes, and cardiovascular disease. FGF21 resistance is one possible explanation, but this theory is not well established. Another possibility is that these pathological conditions induce FGF21 production from different tissue sources, with potentially different signaling pathways, target tissues, or functions. The metabolic functions of FGF21 are clearly extremely complex, and will undoubtedly be further studied. If distinct characteristics and signaling pathways can be revealed between FGF21 derived from different tissues, there could be therapeutic potential in targeting FGF21 production from a particular source in response to a particular stimulus.

## Author Contributions

RS, CM, and LH wrote the manuscript. All authors contributed to the article and approved the submitted version.

## Funding

Funding from the following sources was used for this manuscript: United States Department of Agriculture National Institute of Food and Agriculture (USDA-NIFA, 2019-07916), the University of Washington Royalty Research Fund, and the National Institutes of Health Institute for Diabetes and Digestive and Kidney Diseases (R01DK121370 and F32DK130544).

## Conflict of Interest

The authors declare that the research was conducted in the absence of any commercial or financial relationships that could be construed as a potential conflict of interest.

## Publisher’s Note

All claims expressed in this article are solely those of the authors and do not necessarily represent those of their affiliated organizations, or those of the publisher, the editors and the reviewers. Any product that may be evaluated in this article, or claim that may be made by its manufacturer, is not guaranteed or endorsed by the publisher.

## Abbreviations

ACTH, adrenocorticotropic hormone; Ad-FGF21, adipocyte-derived FGF21; A-FABP, adipocyte fatty acid binding protein; ARC, arcuate nucleus; ATGL, adipose triglyceride lipase; BAT, brown adipose tissue; BCAA, branched-chain amino acid; BMI, body mass index; ChREBP, carbohydrate response element binding protein; DIO2, type II iodothyronine deiodinase; DMH, dorsomedial hypothalamus; FDG, fluorodeoxyglucose; FGF21, fibroblast growth factor 21; FGFR, fibroblast growth factor receptor; GWAS, genome-wide association studies; HDL, high density lipoprotein; Hep-FGF21, hepatocyte-derived FGF21; HFD, high fat diet; HOMA-IR, homeostatic model assessment for insulin resistance; HSL, hormone sensitive lipase; IL6, interleukin-6; IRO, insulin resistant with obesity; ICV, intracerebroventricular; ISO, insulin sensitive with obesity; KLB, beta klotho; KO, knock out; LPL, lipoprotein lipase; MNGIE, mitochondrial neurogastrointestinal encephalomyopathy; MIRAS, mitochondrial recessive ataxia syndrome; NAFLD, non-alcoholic fatty liver disease; NASH, non-alcoholic steatohepatitis; NTS, nucleus of the solitary tract; PGC1α, peroxisome proliferator-activated receptor gamma coactivator-1 alpha; PPARα, peroxisome proliferator-activated receptor alpha; PPARγ, peroxisome proliferator-activated receptor gamma; PVN, paraventricular nucleus; rFGF21, recombinant fibroblast growth factor 21; SCN, suprachiasmatic nucleus; SM-FGF21, skeletal muscle-derived FGF21; SNP, single nucleotide polymorphism; TNF, tumor necrosis factor; UCP1, uncoupling protein-1; VLCKD, very low calorie ketogenic diet; VMH, ventromedial hypothalamus; WAT, white adipose tissue; WT, wild type.
